# Prevention and management of excessive gestational weight gain: a survey of overweight and obese pregnant women

**DOI:** 10.1186/1471-2393-13-10

**Published:** 2013-01-16

**Authors:** W S Leslie, A Gibson, C R Hankey

**Affiliations:** 1 School of Medicine, University of Glasgow, Glasgow Royal Infirmary, 4th Floor, Walton Building, Glasgow Royal Infirmary, 84 Castle Street, Glasgow G4 0SF, UK; 2 Nutrition and Dietetic Department, Lynebank Hospital, Dunfermline, Fife, KY11 4UW, UK

**Keywords:** Pregnancy, Obesity and overweight, Prevention of excess gestational weight gain, Post natal weight retention, Survey

## Abstract

**Background:**

Excessive gestational weight gain is associated with adverse infant, childhood and maternal outcomes and research to develop interventions to address this issue is ongoing. The views of women on gestational weight gain and the resources they would consider helpful in addressing this are however largely unknown. This survey aimed to determine the views of newly pregnant women, living in areas of social disadvantage, on 1) their current body weight and potential gestational weight gain and 2) the resources or interventions they would consider helpful in preventing excessive gestational weight gain.

**Methods:**

A convenience sample of overweight and obese pregnant women living in Fife, UK, were invited to complete a short anonymised questionnaire at their 12 week booking visit.

**Results:**

428 women, BMI>25 kg/m^2^, completed the questionnaire. Fifty-four per cent of respondents were obese (231) and 62% were living in areas of mild to moderate deprivation. Over three-quarters of participants felt dissatisfied with their current weight (81%). The majority of women (60%) expressed some concern about potential weight gain. Thirty-nine percent were unconcerned about weight gain during their pregnancy, including 34 women (19%) who reported having retained weight gained in earlier pregnancies. Amongst those concerned about weight gain advice on physical activity (41%) and access to sports/leisure facilities were favoured resources (36%). Fewer women (12%) felt that group sessions on healthy eating or attending a clinic for individualised advice (14%) would be helpful. “Getting time off work” was the most frequently cited barrier (48%) to uptake of resources other than leaflets.

**Conclusions:**

These data suggest a lack of awareness amongst overweight and obese women regarding excessive gestational weight gain. Monitoring of gestational weight gain, and approaches for its management, should be formally integrated into routine antenatal care. Barriers to the uptake of resources to address weight gain are numerous and must be considered in the design of future interventions and services.

## Background

Obesity is a consequence of energy imbalance, when energy intake exceeds energy expenditure over a prolonged period of time. Most people tend to gain weight gradually in adulthood for numerous reasons including declining physical activity plus small increases in dietary intake. Certain life stages have been identified as critical periods during which weight gain is likely to be more rapid and includes pregnancy [1]. The prevalence of maternal obesity has increased over time and is positively associated with parity, black ethnic group, age and unemployment [2]. Many women attribute their obesity to large weight gains during pregnancy, [3,4], gains which they often never lose. Excess weight gain and failure to lose weight gained during pregnancy are established predictors of long-term obesity [5-7]. Postpartum weight retention increases with greater gestational weight gain [8,9] and gestational weight gain is positively associated with later body mass index (BMI) [10]. Excessive gestational weight gain is associated with increasing deprivation [2] and with adverse infant, childhood and maternal outcomes [2,11,12]. Institute of Medicine guidelines [13] have defined limits for weight gain in pregnancy according to initial BMI. These weight limits vary according to BMI, from 12.7-18.1 kg in those with a BMI<18.5 kg/m [2], 11.3-15.8 kg for those with a healthy BMI (<24.9 kg/m [2]) to 6.8-11.3 kg in the overweight (BMI 25.0-29.9 kg/m [2]) and 4.9 -9.0 kg in the obese. However, while more liberal than previous recommendations, many overweight and obese women still exceed these guidelines [2,13,14].

Pregnant women are potentially receptive to health care advice and may be motivated to implement lifestyle changes [15]. However, currently women’s views on gestational weight gain and its management are largely unknown. The aim of this survey was to determine newly pregnant women’s views on their current body weight and potential gestational weight gain. Opinions on resources that might be considered helpful in preventing excessive weight gain were sought together with any barriers to their use. These data will inform future interventions designed to limit excessive weight gain during pregnancy.

## Methods

This survey was carried out in two hospitals in Fife, Scotland, (Forth Park, Kirkcaldy and Queen Margaret Hospital, Dunfermline) between December 2009 and June 2010. All women attending their booking appointment (at around 12 weeks gestation), whose BMI was in excess of 25 kg/m [2] were invited by their midwife to complete a short questionnaire entitled “weight, health and nutrition during pregnancy”. The questionnaire was devised to address the following research questions:

1) Were women who were already overweight or obese concerned about potential weight gain during their current pregnancy?

2) For those with concerns what opportunities/resources, known to have some impact on energy balance, would be considered useful?

The questionnaire (Additional file 1) comprised eight questions which asked about: 1) number of previous pregnancies (parity) 2) what happened to body weight during these pregnancies; 3) how respondents felt about their current weight and potential weight gain throughout their current pregnancy; 4) which resources would be considered helpful in minimising excessive weight gain. Two questions inquired about potential barriers women felt would prevent them taking advantage of these resources and what, if anything, could help overcome these.

The majority of questions were closed to minimise the burden on respondents. However six questions offered respondents an opportunity to expand on the choices offered and record a resource/option not already listed. Respondents were also invited to record any other comments/views that they might have.

A questionnaire was developed for use in the present study as it was not possible to find a validated questionnaire that addressed the specific area of investigation. Questionnaire content was informed by existing studies of body weight in both pre and post-natal women, by clinical guidelines, and by the clinical experience of the study researcher (AG) [14,16-18].

Formal reliability or validity testing of the survey questionnaire was not performed. However, individual questions and the questionnaires as a whole were reviewed by, and views sought from colleagues working within NHS antenatal clinical service. The calculated Fog index (http://www.thelearningweb.net/fogindex.html) of 7, classified the questionnaire as very readable.

The questionnaire was informally piloted, by reviewing the first 20–30 completed questionnaires to determine any consistent omissions or errors which would have indicated difficulties with any aspect of the questionnaire.

Height, weight, BMI and postcode were recorded by midwives. Postcodes were used to derive a Scottish Index of Multiple Deprivation (SIMD) decile score [19]. SIMD is calculated using key indicators of income, employment, health, education, skills and training, housing, geographic access and incidence of crime. Each post code is allocated an SIMD code.

Questionnaires were completed by respondents on-site at the conclusion of their booking appointment. Completed questionnaires, which were anonymous, were returned in person by respondents to the hospital reception and collected weekly by the study researcher.

### Data analysis

IBM Statistics, version 19, was used to manage and analyse the survey data. Responses were mainly reported descriptively, with chi squared analysis used to determine differences in response by BMI (overweight or obese), and by deprivation category (deciles 1–3, 4–5 and 6–10), factors known to favour excessive gestational weight gain [2].

### Ethical approval

Ethical approval was not required for this study as it was deemed a needs assessment by NHS Fife.

## Results

Between December 2009 and June 2010, 443 women completed the questionnaire. Fifteen respondents were excluded from the analyses, 7 with a BMI below 25 kg /m^2^ and 8 as height and weight were not recorded, thus precluding calculation of BMI.

The mean BMI of the 428 study participants was 31.6 kg/m^2^ (Table 1), with 54% categorised as obese. It was a first pregnancy for 41% (175) of women. The majority of respondents, 60%, resided in areas of deprivation as categorised by SIMD decile 1–5 [19] (Table 1).

**Table 1:** Characteristics of participants

	**Mean**	**n (%)**	**SD**	**Range**
Age (years)	29		6	15 - 50
Weight (kg)	90.6		14.4	54.6 – 145.2
BMI (kg/m^2^)	31.6	428	4.8	25.2 – 49.3
BMI>25 &≤ 30 (kg/m^2^)	27.7	197 (46)	1.4	25.1 - 29.9
BMI>30 (kg/m^2^)	34.9	231 (54)	4.2	30.0 - 49.3
Previous pregnancies	2		2	0-9
SIMD decile *				
1		38 (9)		
2		70 (16)		
3		55 (13)		
4		47 (11)		
5		49 (11)		
6>		169 (40)		

(n=428).

*SIMD decile1-2 = most deprived.

3–5 = moderately deprived.

6>= least deprived.

Sixty percent of women expressed some concern about their current weight (Table 2). Forty three percent of the 252 women with a previous pregnancies reported they had not lost the weight gained during previous pregnancies. However, 19% (49) of these women felt that despite their previous weight gain and retention they were comfortable with their current weight. Of 39 women who reported “gaining a lot of weight but not losing it in a previous pregnancy” 32, (82%) currently had a BMI >30 kg/m^2^.

**Table 2:** Opinions on current weight and potential gestational weight gain

	**All respondents (n=428)**	**BMI > 25 & < 29.9 kg/m**^**2 **^**(n=197)**	**BMI *>*30 kg/m**^**2 **^**(n=231)**	**SIMD 1–3 (n=163)**	**SIMD 4–5 (n=96)**	**SIMD 6–10 (n=169)**
**Views on current weight**						
Comfortable	173 (19)	69 (35)	12 (5)	34 (21)	14 (15)	33 (19)
Probably a bit heavy	148 (35)	81 (41)	67 (29)	52 (32)	39 (41)	57 (34)
Been trying to watch weight already	107 (25)	28 (14)	79 (34)	42 (26)	27 (28)	38 (22)
Not happy	86 (20)	17 (9)	69 (30)	33 (20)	15 (16)	38 (22)
No response	6 (>1)	2 (>1)	4 (>1)	2 (>1)	1 (>1)	3 (2)
**Opinions concerning weight gain in previous pregnancies**	(n=252)					
Acceptable weight gain & return to pre pregnancy weight	85 (37)	46 (47)	39 (29)	28 (30)	51 (43)	40 (44)
Acceptable weight gain but did not return to pre-pregnancy weight	71 (30)	25 (26)	45 (33)	28 (30)	33 (28)	25 (27)
Gained a lot of weight but lost it after having the baby	39 (17)	20 (20)	19 (14)	14 (15)	22 (18)	13 (15)
Gained a lot of weight but did not lose it after having the baby	39 (17)	7 (7)	32 (24)	22 (24)	14 (11)	8 (9)
No response	19 (8)	1 (<1)	10 (14)	10 (10)	6 (10)	5 (5)
**Views on potential weight gain in current pregnancy **(n=428)						
Not concerned	162 (39)	95 (50)	65 (28)	62 (38)	100 (38)	61 (36)
Expect to gain some weight but don’t want it to be too much	199 (47)	77 (40)	120 (52)	76 (47)	123 (48)	84 (49)
Really worried about gaining too much weight	57 (13)	16 (9)	41 (18)	22 (14)	35 (13)	18 (11)
No response	10 (<1)	12 (1)	2 (<1)	4 (<1)	3 (<1)	6 (3)

Data are values & (%).

Thirty nine percent (n=162) of participants reported being unconcerned about potential weight gain in their current pregnancy, and this included 34 (22%) that had not lost weight gained in previous pregnancies. Responses differed little according to deprivation index. However, comparison by BMI category of participants views on their current weight showed obese respondents to be less happy with their current weight than those who were overweight (p<0.001).

Among those who expressed concern about potential weight gain, advice on physical activity was most frequently cited as the resource which would be most helpful in minimising weight gain followed by access to sports/leisure facilities (Table 3). Fewer women, 12%, indicated that other group sessions on healthy eating or attending a clinic for individualised advice (14%) would be helpful.

**Table 3:** Opinions on resources which may help minimise weight gain in pregnancy and perceived barriers to their uptake among women concerned about weight gain

**Resources**	**All (n=256)**	**BMI > 25 & < 29.9 kg/m**^**2 **^**(n=95)**	**BMI > 30 kg/m**^**2 **^**(n=161)**	**SIMD 1–3 (n=98)**	**SIMD 4–5 (n=56)**	**SIMD 6–10 (n=102)**
Advice on physical activity	107 (42)	37 (39)	70 (44)	37 (38)	24 (43)	46 (45)
Access to sports/leisure facilities	92 (36)	41 (43)	52 (32)	38 (39)	13 (23)	42 (41)
Leaflets on healthy eating in pregnancy	93 (36)	37 (39)	57 (35)	38 (39)	15 (27)	41 (40)
Leaflets on healthy eating	58 (23)	23 (24)	36 (22)	23 (24)	14 (15)	22 (22)
Attending a clinic to get advice targeted to me	33 (13)	10 (11)	23 (14)	12 (12)	3 (5)	18 (18)
Attending a group about healthy eating	31 (12)	12 (13)	19 (11)	9 (9)	8 (14)	14 (14)
Attending a class to learn how to cook healthy meals	26 (10)	13 (14)	13 (8)	8 (8)	7 (13)	11 (11)
Supermarket tour	3 (1)	1 (1)	2 (1)	1 (1)	0	2 (2)
**Perceived barriers to uptake of resources**						
Getting time of work	120 (47)	50 (53)	70 (44)	36 (37)	31 (55)	53 (52)
Other children to look after	84 (33)	31 (33)	53 (33)	37 (38)	19 (34)	28 (27)
Too shy to go into a new situation	33 (13)	10 (11)	23 (14)	18 (18)	6 (11)	9 (9)
Cost of travel	15 (6)	6 (6)	9 (6)	7 (7)	2 (4)	6 (6)
**What would help attendance at classes/appointments?**						
Held near my home	111 (43)	46 (48)	66 (41)	45 (46)	27 (48)	40 (39)
If they were held in evening or at weekends	119 (46)	45 (47)	74 (46)	42 (43)	25 (45)	52 (51)
If I could bring a partner or friend	65 (25)	22 (23)	43 (27)	38 (39)	9 (16)	18 (18
If they were not held in a clinic or hospital	8 (31)	5 (5)	3 (2)	4 (4)	2 (4)	2 (2)

The most frequently cited barrier to accessing resources other than leaflets was “getting time off work” (48%). The provision of sessions nearer to participant’s homes and/or at weekends/evenings was cited by respondents as a way of challenging this barrier. There were no “other responses” recorded, for any questions.

Responses regarding resources did not differ by BMI category or deprivation index.

## Discussion

The majority of women who participated in the present survey expressed some concern over the weight gain that might be experienced during their current pregnancy. Previous research carried out amongst a diverse sample of mothers also found many had concerns about gestational weight gain [20,21].

In the present survey women who expressed concern about weight gain reported more interest in increasing their physical activity than receiving targeted dietary advice. Physical activity is something known to have health benefits during pregnancy [22] and specific practical advice to increase activity is available [23]. However evidence suggests that interventions which focus solely on increasing physical activity during pregnancy have little effect on minimising excessive gestational weight gain, as activity levels often fall, the decrease in activity persisting at 6 months post partum [24,25]. It remains an area where further research is justified [26]. Respondents may have viewed physical activity as being relatively easy to incorporate into daily life, however in practice this may not be the case. Compliance with the physical activity component of a recent study was reported as difficult to sustain [27] due to self reported minor ailments leading women to refrain from activity. Numerous barriers to engaging in physical activity during pregnancy have also been cited including: work, lack of motivation and lack of time [22]. It is unlikely that interventions that rely solely on changes to physical activity would be effective in minimising excessive gestational weight gain. Successful weight management interventions, in non-pregnant individuals, require changes to both diet and physical activity and these principles would apply to the prevention of excessive gestational weight [18,28].

In the UK elements of antenatal care such as prenatal “birth groups” have, for many years, traditionally been delivered in a group format. In the present study both group based and one to one interventions were not popular. A low intensity intervention targeting diet and physical activity, with minimal face to face contacts achieved an 82% retention rate six months post-partum. Study outcomes were encouraging with more overweight women returning to their pre-pregnancy weight, than those in receipt of usual care [29]. However, 60% of women who were eligible and contactable either declined to participate or failed to attend. Uptake to this study was therefore only slightly better than in other research which used a group approach [14]. The low uptake to either form of intervention for weight management during pregnancy suggests other barriers that may preclude participation. It may be that contrary to opinion pregnancy is in fact not a time when women are receptive to change. Anderson [30] described pregnancy as a time when good dietary choices are maintained, but not as an opportunity for implementing dietary or other health changes. The present survey findings appear to support this assertion. However, it must be noted that the present data represent only the views of those who chose to participate in the survey. Non-participants may have held opposing views.

Getting time off work was the most frequently reported barrier to accessing resources to minimise gestational weight gain. This concurs with other research which explored the reasons women declined to participate in an intervention to address obesity in pregnancy [31]. This study also found that the location of services was also a barrier to participation. In the present survey women indicated they may be more likely to attend a service that was held near to their home and out-with working hours. These barriers may be difficult to address within the current NHS service provision. However, increasing opening hours for general practitioners surgeries/health centres could circumvent these barriers; some are now open out-with conventional working hours. Successful uptake may also be facilitated by use of a low intensity approach similar to that used by Phelan et al. [29], where many of the barriers to participation reported by study participants, were overcome.

Evidence regarding the efficacy of interventions to reduce excessive gestational weight gain is conflicting. One review of interventions concluded that the quality of published trials was insufficient to allow evidence based recommendations for clinical practice to be developed [17]. Two more recent systematic reviews were more positive, both finding a 2 kg weight difference at six months partum, and one also finding a significant fall in the incidence of caesarean section in those women in receipt of a dietary intervention [23,24].

Around 40% of women in the present survey, including those who had not lost weight gained in previous pregnancies, reported being unconcerned about potential weight gain during their pregnancy, a finding that is not unique. Earlier research carried out to elicit the views of pre and post-natal women on gestational weight gain found that in general the women interviewed were unconcerned about potential weight gain with many assuming that weight loss would occur in the post natal period as a result of breast feeding [32].

Many women do not acknowledge that their weight is an issue [14]. This may, in part, be due to the “normalisation” of obesity among the general population [31]. Comparison of perceptions of weight from two population surveys in the UK found that increasingly, overweight and obese individuals fail to recognise their weight as a cause for concern [31] This was also found to be the case in the United States [32].

Attitudes regarding gestational weight gain may reflect a lack of awareness of the potential implications of excessive gestational weight gain [33]. The present study did not explore women’s beliefs regarding the impact of gestational weight gain, however previous research suggests that while women acknowledge that insufficient weight gain may adversely affect their child few recognise the impact that excessive weight gain could have on infant health [21,22]. Unawareness of the potential consequences of excessive gestational weight gain on both maternal and infant health suggests that engaging women in interventions to prevent excessive gestational weight gain is likely to be difficult [21]. If weight is not viewed as a concern by patients, then weight management interventions are unlikely to be seen as personally relevant [31] and destined to failure.

Lack of concern regarding gestational weight gain may also reflect a lack of emphasis on lifestyle issues by the healthcare professionals delivering care. Clinical guidelines [18,28] advocate that as part of their antenatal care women should routinely receive advice on becoming physically active and consuming a healthy diet. Midwives have acknowledged that raising the issue of a pregnant women’s weight is something they find difficult, and often causes offence [14]. The CMACE Project reported that evidence of discussion regarding the risks of obesity in pregnancy was documented in less than one-fifth of cases audited. It is likely that issues of weight are not raised or addressed with many women [22]. Lack of knowledge among health professionals regarding appropriate gestational weight gain has also been reported [21] and is likely to reflect the lack of formal recommendations in the UK [22].

In the UK the regular measurement of maternal body weight during pregnancy is not advocated, and is therefore measured infrequently [15]. There is reluctance among women to have their weight measured regularly as they do not wish to know how much weight has been gained [21]. However, regular measurement of body weight could highlight to patients that changes in weight are important during pregnancy, and also offer midwives the opportunity to raise and discuss diet and physical activity. Midwives themselves have reported that women are more likely to engage when they consider weight measurements to be part of routine care and that integrating weight monitoring into routine care avoids women feeling stigmatised [34]. New services in Australia have shown that midwives, when trained, have integrated issues related to weight gain into usual care [14]. In the UK midwives have expressed a need for training specific to obesity to enable them to provide targeted advice [34,35]. Since this survey was completed a UK wide campaign highlighting the issue of obesity and pregnancy has been launched [36].

### Study strengths and limitations

The primary aim of the present study was to ascertain service user’s views on resources they may find helpful in managing gestational weight gain. The questionnaire focussed solely on this issue and did not explore women’s knowledge and understanding of the possible effects of excessive gestational weight gain on maternal and infant health which may have provided greater insight into the lack of concern regarding weight gain expressed by 40% of respondents.

One strength of this study is that it was carried out in clinical practice and engaged women at the point they are first utilising clinical services (their week 12 booking visit) and are at a point when they may be thinking about lifestyle issues such as diet, health, body weight and their overweight or obesity. The present findings reflect women’s current thoughts, and are therefore free of the shortcomings of data collected both prospectively and retrospectively.

The choice of resources given in the questionnaire to potentially assist in the management of gestational weight gain was limited, and based on resources and strategies already advocated in clinical guidelines [18,28]. The option for respondents to propose other resources was also given but was not utilised. We acknowledge that this may reflect reluctance by respondents to articulate their own choices and recognise there may be other resources out-with those cited in the present survey which women would be willing to utilise. A greater insight into patient’s views may have been seen with the inclusion of a “value-based” question, which could have quantified opinions of the suggested resources further.

Validation of the questionnaire would have strengthened the findings of the study, but was not carried out due to time and resource constraints. As weight and height measurements are not routinely made in Fife it was not possible to compare the study respondents with the overall sample frame. Ethnicity, which is associated with maternal obesity, and may also influence views on potential resources, was not documented in the present survey. The survey’s location, Fife, Scotland, has a small black and ethnic population estimated at 1.5% in 2001, [37] thus it is unlikely that any survey in this area would include a large proportion of ethnic groups.

## Conclusion

Population strategies for the prevention and treatment of obesity are a priority and pregnancy and post partum are well recognised as risk periods for the onset and aggravation of overweight and obesity [1,28,38]. Pregnant women are therefore an appropriate target population for interventions to avoid excessive gestational weight gain. Research on approaches for the management of weight gain at this time is a growing area, reflecting its importance and considerable implications for maternal and infant health. There is only scant research on the concerns, if any, of pregnant women regarding weight gain during pregnancy and the resources they may value in trying to address this. The present study provides some insight into the thoughts and perceived needs of a largely deprived sample of overweight and obese pregnant women, many of whom were unconcerned about gestational weight gain. Our findings highlight a need to formally integrate discussions on weight gain and its management into routine antenatal care. Barriers to the uptake of resource to address weight gain are numerous but must also be considered in the design of future interventions and services. Training and education in weight management is required before midwives feel adequately equipped to raise and address the issue of gestational weight gain with their patients.

## Competing interests

The authors declare that they have no competing interests.

## Authors’ contribution

WL designed the study, carried out data analysis and contributed to the manuscript. AG designed the study, performed data collection and contributed to the manuscript. CH designed the study, contributed to data analysis and drafted the manuscript. All authors have critically reviewed and approved the final manuscript.

## Pre-publication history

The pre-publication history for this paper can be accessed here:

http://www.biomedcentral.com/1471-2393/13/10/prepub

## Supplementary Material

Additional file 1 Weight, Health and Nutrition During Pregnancy. Click here for file
