# Supplementary material for: Prevention and management of excessive gestational weight gain: a survey of overweight and obese pregnant women
Source: BMC Pregnancy Childbirth. 2013 Jan 16;13:10. doi: 10.1186/1471-2393-13-10 (PMC3616937; doi:10.1186/1471-2393-13-10)
Supplement: Additional file 1 — Weight, Health and Nutrition During Pregnancy. [file 1471-2393-13-10-S1.doc]

**Weight, Health and Nutrition During Pregnancy**

**Patient: Age.......... SIMD code...............**

**Height.............Weight.............**

We are interested in finding out the best approach to help women not to put on too much weight during pregnancy. The information we get from this questionnaire will allow us to understand what women want and need during their pregnancy and help us plan our services. All information will be kept confidential.

We would be grateful if you could spend a few minutes answering the questions then give the completed form to the staff at reception.

1. Have you had any previous pregnancies? *Please tick one box only*

Yes  No  If yes, how many? …..

2. What happened to your weight during your previous pregnancies?

*Please tick one box only*

 Acceptable weight gain and returned to pre-pregnancy weight

 Acceptable weight gain but did not lose again after having baby

 Gained a lot of weight but lost it after having baby

 Gained a lot of weight but did not lose it after having baby

3. How do you feel about your weight at the moment?

*Please tick one box only*

 Comfortable

 Probably a bit heavy

 Been trying to watch weight already

 Not happy

 Other (please comment)...................................................................

4. Do you have any concerns about putting on too much weight during this pregnancy? *Please tick one box only*

 Not concerned, expect to gain weight

(if you tick this response do not fill in any more of the questionnaire)

 Expect to gain some weight but don’t want it to be too much

 Really worried about gaining too much weight

 Other (please comment) …………………………………………...

5. What do you feel would help you to prevent putting on too much weight during this pregnancy? *(please tick as many boxes as necessary)*

 Leaflets on healthy eating

 Leaflets about healthy eating in pregnancy

 Attending a group about healthy eating

 Supermarket tour

 Attending a class to learn how to cook healthy meals

 Attending a clinic to get advice targeted to me

⁭ Advice on physical activity

 Access to sports/leisure facilities

 Other (please comment) ………………………………………….

6. What would stop you attending for help to prevent putting on too much weight? *(please tick as many boxes as necessary)*

 Getting time off work

 Cost of travel

 Too shy to go into a new situation

 Other children to look after/ other responsibilities

 Other (please comment) ………………………………………….

7. What would help you to be able to attend classes and appointments?

*(please tick as many boxes as necessary)*

 Held near my home

 If I could bring my partner or a friend

 If they were in the evening or at weekends

 If they were not held in a clinic or hospital

 Other (please comment) ………………………………………….

8. Any other comments? ……………………………………………….

Thank you very much for your help.
